# Supplementary material for: Giant activity-induced elasticity in entangled polymer solutions
Source: Nat Commun. 2025 Jun 12;16:5305. doi: 10.1038/s41467-025-60210-9 (PMC12163070; doi:10.1038/s41467-025-60210-9)
Supplement: Supplementary file 1 — Supplementary Information [file 41467_2025_60210_MOESM1_ESM.pdf]

Supplementary Information: Giant Activity-Induced Elasticity in Entangled Polymer Solutions

Davide Breoni,[1] Christina Kurzthaler,[2, 3, 4] Benno Liebchen,[5] Hartmut Löwen,[1] and Suvendu Mandal[1, 5, *]

[1] *Institut für Theoretische Physik II: Weiche Materie, Heinrich Heine-Universität Düsseldorf, Universitätsstraße 1, 40225 Düsseldorf, Germany*
[2] *Max Planck Institute for the Physics of Complex Systems, Nöthnitzer Straße 38, 01187 Dresden, Germany*
[3] *Center for Systems Biology Dresden, Pfotenhauerstr. 108, 01307 Dresden, Germany*
[4] *Cluster of Excellence, Physics of Life, TU Dresden, 01062 Dresden, Germany*
[5] *Technische Universität Darmstadt, Karolinenplatz 5, 64289 Darmstadt, Germany*

Contents

I. Validity of the Green-Kubo formalism 1
II. Frequency-dependent viscoelastic properties 2
III. Time-dependent shear stress 5
IV. System equilibration 5
V. Primitive path analysis and topology 6
VI. Polymer conformation and entanglement length 6
VII. Viscoelasticity of less entangled systems 6
VIII. Single-mode relaxation of $G(t)$ at long times 8
IX. Number of entanglement points and active tube model 8
References 10

I. Validity of the Green-Kubo formalism

Here, we show in detail that the criteria for applying Green-Kubo relations far from equilibrium [1], clearly apply to our work.

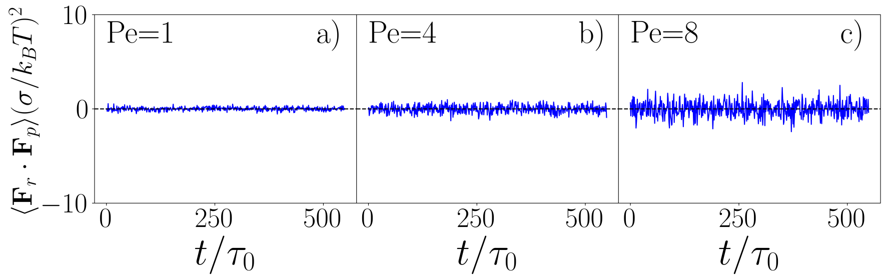

Figure S1. **Correlation between Brownian and self-propulsion forces.** The time evolution of the correlation between fluctuating Brownian forces and self-propulsion forces, $\langle \mathbf{F}_r \cdot \mathbf{F}_p \rangle$. The correlation remains close to zero on average, as indicated by the black dashed line, confirming the statistical independence of thermal fluctuations and active driving forces.

* suvendu.mandal@pkm.tu-darmstadt.de

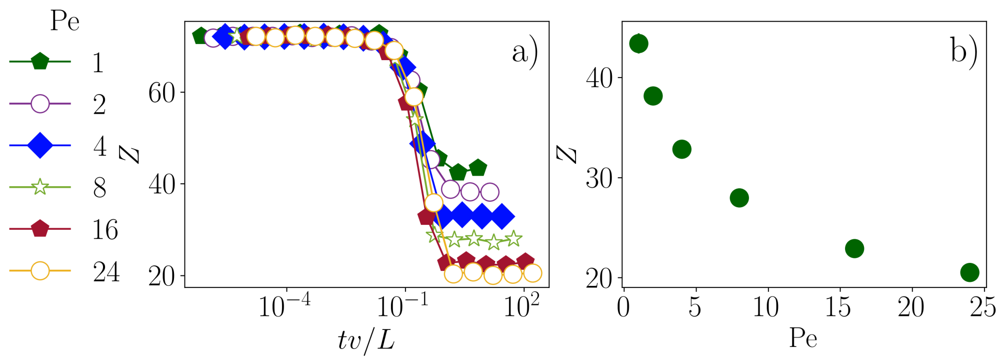

Figure S2. **Time evolution and steady-state behavior of entanglement points across Péclet numbers.** (a) Time evolution of the number of entanglement points, Z, for various Péclet numbers (Pe) at a fixed polymer length $L = 1450\sigma$. (b) Number of entanglement points at the steady state as a function of Pe, illustrating how increasing activity affects polymer entanglement points.

1. **Decoupling of activated and fluctuating degrees of freedom:** In our model, the fluctuating Brownian forces $\mathbf{F}_r$, which determine the thermal noise of the system, are Gaussian white noise and remain statistically uncorrelated with the self-propulsion forces $\mathbf{F}_p$ (activated degrees of freedom) [see Fig. S1]. This statistical decoupling ensures that the dynamics of the activated forces are not influenced by thermal fluctuations.

2. **Stability of the steady state:** Han et al. [1] demonstrated that active systems perturbed slightly from their steady state relax back to this steady state rather than to equilibrium. Consistent with this principle, our active polymer system operates in a stable non-equilibrium steady state. Specifically, we observe that:

   - Stress fluctuations average to zero in the steady state [see Fig. S8].
   - The number of entanglements saturates after the disengagement time $\tau_{\text{eff}} = L/v$, where L is the polymer length and v is the self-propulsion velocity [see Fig. S2].

   These observations indicate the stability of the steady state.

3. **Isotropy of the system:** We compute the radial distribution function (RDF) and confirm that our system remains isotropic, even at high Péclet numbers [see Fig. S3]. To understand the evolving orientational order, we have further calculated the nematic order parameter $P_2(n) = \frac{1}{2}(3\langle\cos^2(\vartheta_n)\rangle - 1)$, where $\langle\rangle$ indicates an ensemble average and ϑ_n is the angle between the $z-$axis and the vector $\mathbf{R}_n$ between beads separated by n bonds. One expects that $P_2(N_p - 1) = 0$ for randomly oriented chains and $P_2(N_p - 1) = 1$ for perfectly aligned chains [2]. It turns out that our systems show $P_2(N_p - 1) \approx 0$ [see Fig. S4(a)], which further validates that our system remains isotropic.

   Here, we would like to emphasize that anisotropy in the RDF is a prerequisite for non-zero shear stress [3, 4]. In contrast, the isotropy in the RDF observed in our system ensures that shear stress fluctuations average to zero [see Fig. S8], further validating the assumptions of the Green-Kubo framework.

4. **Decay of the spatial velocity-velocity correlations:** To confirm the final condition, we calculate the velocity autocorrelation functions [see Fig. S4(b)]. Analyzing the spatial velocity correlations, we find that they decay beyond the polymer (monomer) diameter, consistent with a decay faster than r^{-3} in three dimensions. This result satisfies the condition that velocity correlations must decay sufficiently rapidly, as outlined by Han et al. [1].

Finally, we note that Green-Kubo relations have been successfully applied to other active systems, such as active Brownian particles (ABPs), where transport coefficients are derived using non-equilibrium steady-state correlation functions as the reference state [5]. Furthermore, a Green-Kubo relation has been devoloped to expolore the odd diffusivity and the odd viscosity in non-equilibrium set-ups [6–8].

II. Frequency-dependent viscoelastic properties

To characterize the viscoelastic properties of active entangled polymer solutions, we compute the storage modulus $G'(\omega)$ and loss modulus $G''(\omega)$ from the stress relaxation modulus $G(t)$ via Fourier transformation, following standard

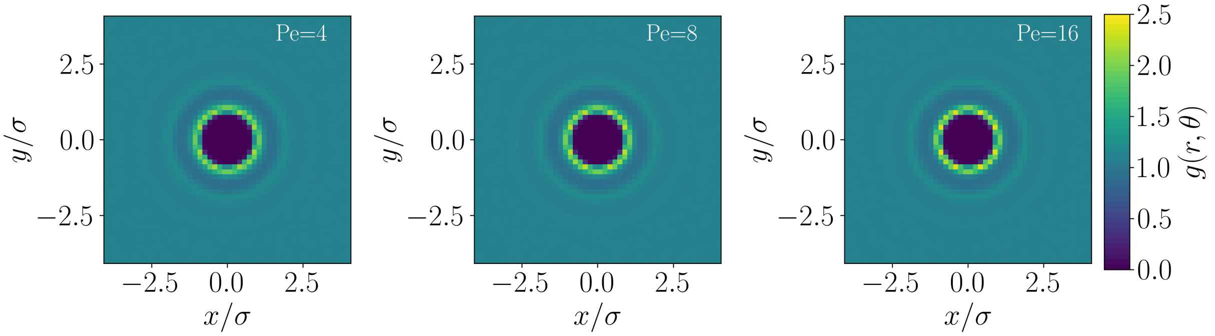

Figure S3. **Isotropy of the radial distribution function across varying Péclet numbers.** The radial distribution function $g_{xy}(r, \vartheta)$ in the xy-plane for various Péclet numbers (Pe) shows that the system remains isotropic, even with increasing activity.

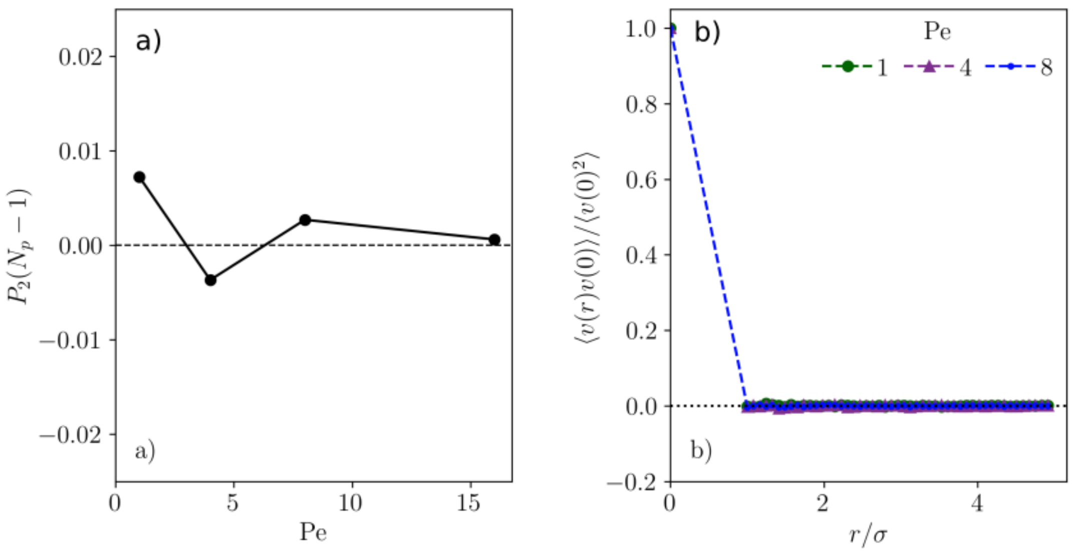

Figure S4. **Orientational order and velocity correlations in active polymers across varying Péclet numbers.** (a) The orientational order parameter $P_2(N_p - 1)$, where $N_p = 725$ is the number of monomers per chain, as a function of the Péclet number Pe. The data show how the orientational order evolves with increasing activity, reflecting the degree of alignment of polymer chains under active driving. Despite increasing Pe, the system maintains a relatively low orientational order (≈ 0), indicating that alignment does not develop even at high activity levels. (b) The spatial velocity correlation $\langle \mathbf{v}_i(r) \cdot \mathbf{v}_i(0) \rangle$, where $\mathbf{v}_i = \mathrm{d}\mathbf{x}_i/\mathrm{d}t$, as a function of Péclet number Pe. The results show that the velocity correlations decay rapidly, vanishing once the displacement distance r exceeds the monomer diameter. This rapid decay indicates that the system lacks long-range velocity correlations, even at high activity levels.

methods in polymer physics [9]. To address this, we have computed $G'(\omega)$ and $G''(\omega)$ from $G(t)$ using the standard relations:

$$G'(\omega) = \omega \int_0^\infty G(t) \sin(\omega t)\, \mathrm{d}t,$$
$$G''(\omega) = \omega \int_0^\infty G(t) \cos(\omega t)\, \mathrm{d}t.$$

Here, $G'(\omega)$ represents the storage modulus, which quantifies the system's elastic response, and $G''(\omega)$ characterizes the viscous dissipation. The frequency-dependent shear moduli are ideally suited to distinguish elastic behavior (dominant at high frequencies, $G' > G''$) from viscous behavior (dominant at low frequencies, $G' < G''$) in polymer systems [see Fig. S5(a)]. Our results show the following features:

1. **Crossover between G' and G'' at low frequencies:** The crossover frequency, where G' and G'' intersect, reflects the transition from viscous to elastic behavior and marks the effective disengagement time τ_{eff} [see Fig. S5(a)]. Our results confirm that $\tau_{\text{eff}} \sim L/v$, validating the key scaling predictions [see Fig. S5(b)].

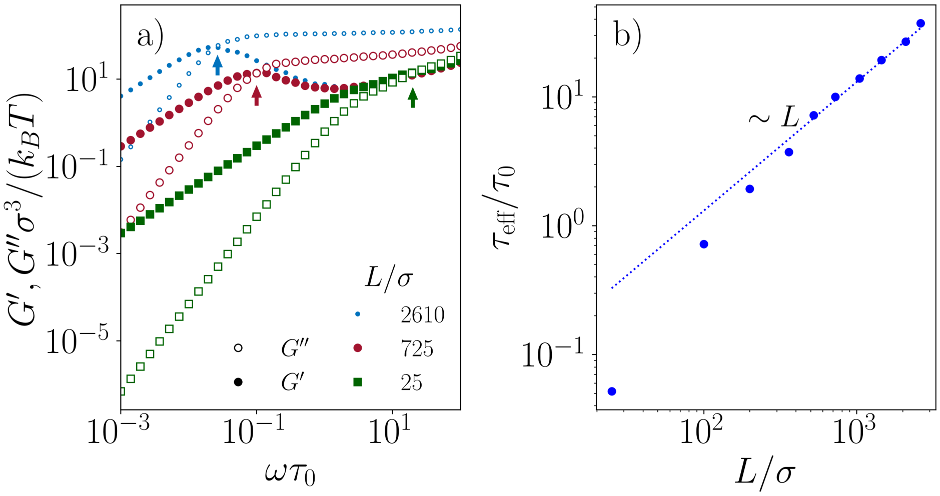

Figure S5. **Frequency-dependent viscoelastic properties and polymer disengagement time at a fixed Péclet number, Pe=16.** (a) Frequency-dependent storage modulus G' (empty symbols) and loss modulus G'' (open symbols) as functions of frequency ω. The crossover point (indicated by markers) at which G' and G'' intersect reveals the timescale at which active polymers escape their confinement within entanglement tubes. (b) The extracted disengagement time $\tau_{\rm eff}$, plotted as a function of polymer length L, follows the scaling relation $\tau_{\rm eff} \sim L/v$, where v is the self-propulsion speed.

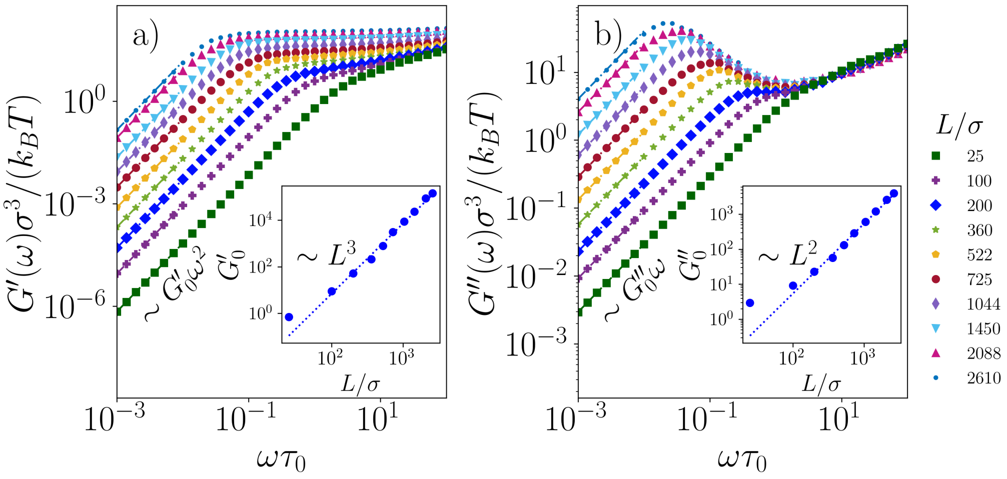

Figure S6. **Frequency-dependent viscoelastic properties of active entangled polymer solutions at a fixed Péclet number, Pe=16.** (a) Storage modulus G' and (b) loss modulus G'' as functions of frequency ω. At low frequencies, G' and G'' exhibit scaling behavior characteristic of viscous-dominated relaxation. The inset in (a) highlights the low-frequency prediction of the storage modulus $G_0' = G'(\omega \to 0)$, while the inset in (b) shows the corresponding prediction for the loss modulus $G_0'' = G''(\omega \to 0)$. The results demonstrate the transition from viscous to elastic behavior, revealing the hallmark features of entangled active polymer systems.

2. **Low-frequency scaling:** At low frequencies ($\omega \to 0$), $G'(\omega)$ scales as $\sim \omega^2$. This scaling arises from the expansion $\sin(\omega t) \approx \omega t$, leading to $G'(\omega) \sim \omega^2 \int_0^\infty G(t) t\, dt \sim \omega^2 G_0 \tau_{\rm eff}^2 \sim \omega^2 L^3/v$, where L is the polymer length and v is the self-propulsion speed [see Fig. S6(a)]. Similarly, $G''(\omega)$ scales linearly as $G''(\omega) \sim \omega \int_0^\infty G(t)\, dt \sim \omega G_0 \tau_{\rm eff} \sim \omega L^2$, consistent with the series expansion $\cos(\omega t) \approx 1$ [see Fig. S6(b)].

3. **Elastic plateau at intermediate frequencies:** At intermediate frequencies, $G'(\omega)$ displays a plateau, a hallmark of entangled polymer systems [see Fig. S6(a)]. The plateau modulus G_0 is extracted from the minimum in the loss-to-storage ratio, $\tan\delta = G''/G'$ [see Fig. S7(a)]. The results confirm the scaling $G_0 \sim L$ at a fixed Péclet number, as shown in Fig. S7(b).

These results provide a comprehensive characterization of the viscoelastic properties of active entangled polymer

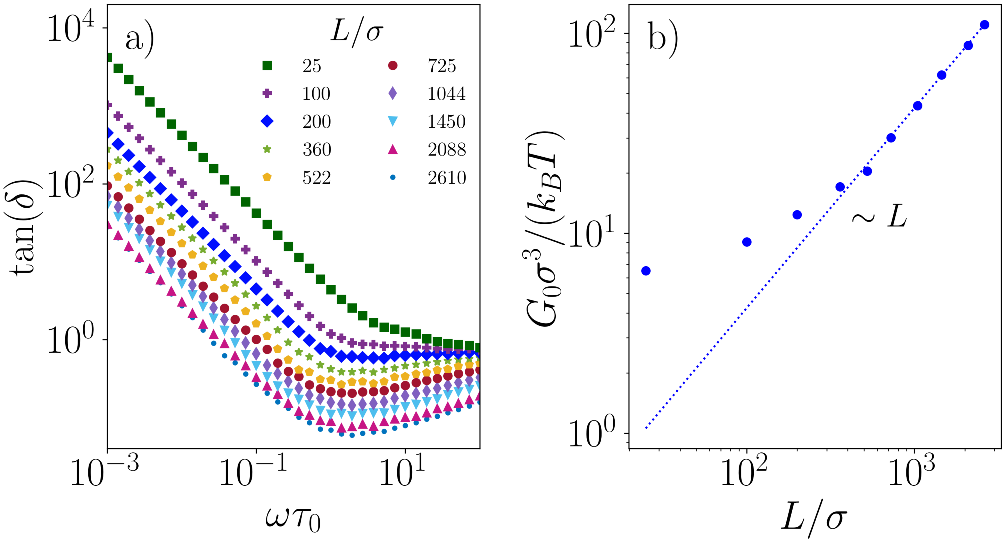

Figure S7. **Extraction of the plateau modulus G_0 from the minimum of the loss-to-storage ratio at a fixed Péclet number, Pe=16.** (a) The minimum in $\tan\delta = G''/G'$ occurs at a frequency near the center of the elastic plateau. The storage modulus G' at this frequency provides a robust estimate of the plateau modulus G_0. (b) The extracted G_0 scales linearly with polymer length L, confirming the prediction $G_0 \sim L$ at a fixed Péclet number.

solutions and validate the scaling laws derived in our theoretical framework. The additional insights link the stress relaxation modulus $G(t)$ to the frequency-dependent shear moduli $G'(\omega)$ and $G''(\omega)$, clarifying the dynamic behavior of active polymer systems.

III. Time-dependent shear stress

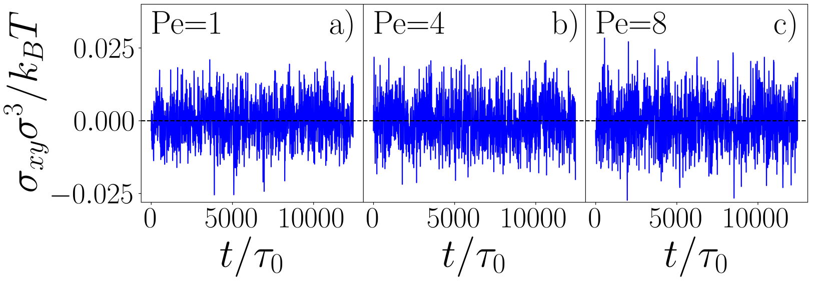

Figure S8. **Time evolution of shear stress for different Péclet numbers.** Time-dependent shear stress $\sigma_{xy}(t)$ for different Péclet numbers: (a) Pe = 1, (b) Pe = 4, and (c) Pe = 8. The figure illustrates how shear stress evolves over time under varying Péclet numbers.

Our simulations of the off-diagonal stress component, $\sigma_{xy}(t)$, show that it fluctuates around zero over time [Fig. S8]. This contrasts with externally-driven systems, such as shear flow between two plates, where a finite, non-zero stress is observed. Despite the presence of active forces, our system does not exhibit the sustained shear stress characteristic of externally-driven non-equilibrium systems; instead, internal stresses fluctuate dynamically.

IV. System equilibration

A well-known challenge in the field of entangled polymer physics has been the excessively long timescales required to reach equilibrium, with relaxation times scaling as the cube of the polymer length, i.e., $\sim L^3$. To circumvent the need for exceedingly lengthy simulations, we employ a highly efficient approach known as the double-bridging hybrid

(DBH) bond-swapping algorithm, in conjunction with core softening techniques as outlined in Dietz et al.'s work [10]. The DBH algorithm operates by executing Monte Carlo (MC) moves to swap bonds and angles within the context of molecular dynamics simulations [Fig. S9]. Notably, this technique allows for the exchange of entire strands of polymers, a capability not available in standard molecular dynamics simulations. As a result, it substantially reduces the relaxation time, transitioning from the daunting $\sim L^3$ scaling to a much more manageable $\sim L$.

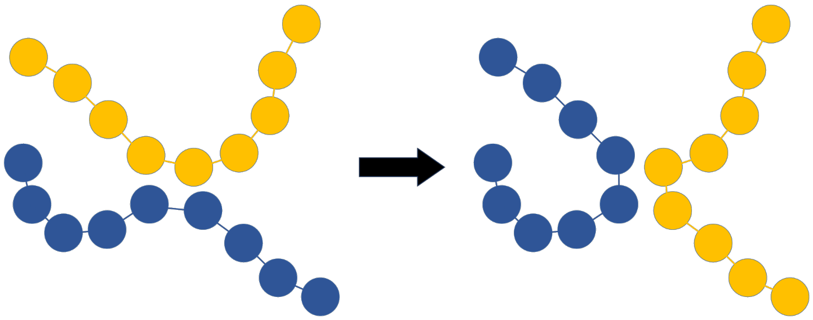

Figure S9. **Bond exchange between polymer chains.** Representation of a double-bridging hybrid Monte Carlo move, demonstrating the exchange of bonds between separate polymer chains.

V. Primitive path analysis and topology

To explore the system's topology, we employ the Z1+ algorithm developed by M. Kröger [11]. The Z1+ algorithm iteratively simplifies the initial polymer configuration based on entanglement point positions, thus revealing the essential topological structure of the primitive paths [see Fig. S10(a)]. It begins by examining sets of three consecutive nodes along each polymer, initially defined by monomer positions. It evaluates the area enclosed by the triangle formed by these nodes, accounting for potential obstacles defined by intersecting paths. After multiple iterations, when further area reduction becomes unattainable, the resulting nodes represent the system's topological entanglement points. The average number of entanglement points, denoted as Z, is calculated as the mean number of nodes per path, while L_{pp} signifies the average path length. Figure S10(b) presents the final primitive path of a tracer polymer and its neighboring paths obtained using the Z1+ algorithm from a simulation configuration.

VI. Polymer conformation and entanglement length

Our investigation of the end-to-end distance R_{ee} of polymer chains at long times reveals a striking consistency: irrespective of the applied Péclet number (Pe), the system exhibits a common scaling law, $R_{ee} \sim L^{1/2}$, similar to ideal polymer solutions [see Fig. S11(a)]. Intriguingly, the prefactor of this scaling relation steadily decreases with increasing Pe, reminiscent of a coil-to-globule transition, observed in dilute active flexible polymer solutions [12]. However, in our complex, densely entangled networks, a true globule-like structure does not occur; instead, the $L^{1/2}$ scaling exponent remains valid across all Pe values, highlighting a consistent entangled behavior in response to activity.

In Fig. S11(b), we explore the temporal evolution of the end-to-end distance for various polymer lengths L at a fixed Péclet number Pe = 8. Given that the $R_{ee} \sim L^{1/2}$ scaling remains valid across for all Péclet numbers, we anticipate the normalized R_{ee}/R_{ee}^0 to collapse at long times ($tv/L \gg 1$), as depicted in Fig. S11(b). Furthermore, we note a gradual reduction in R_{ee}, persisting until $tv/L \sim 1$. In contrast, we observe a 10% increase in the contour length of the primitive path (L_{pp}) at intermediate times ($tv/L \sim 0.1$), as illustrated in Fig. 1(c) of the main text. As a consequence, the entanglement length (N_e/N_e^0) is expected to exhibit a 30% decline at $tv/L \sim 0.1$ before ultimately reaching a saturation value at long times [see Fig. S11(c)].

VII. Viscoelasticity of less entangled systems

One could anticipate that the larger values of $G(t)$ at higher activities may result from effectively increased monomeric friction. To demonstrate the unique nature of the stress plateau enhancement due to activity in en-

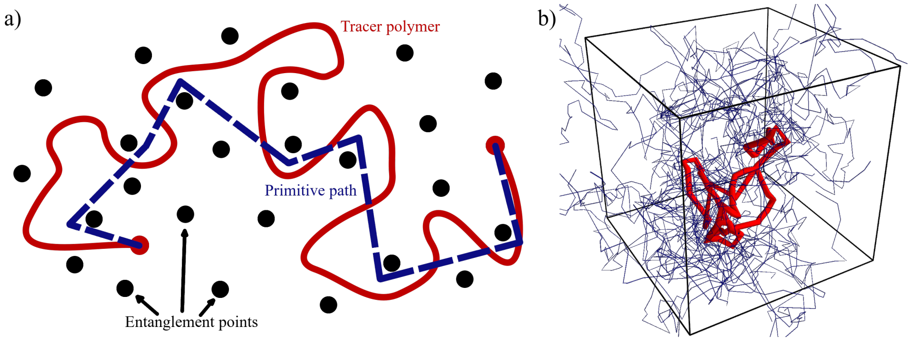

Figure S10. **Visualization of the Z1+ algorithm and primitive path analysis.** (a) Schematic representation illustrating the operation of the Z1+ algorithm. The primitive path (blue) relative to a tracer polymer (red) is depicted, with entanglement points (black) representing obstacles posed by other polymers. (b) Snapshot from a simulation displaying the primitive paths of a tracer polymer (red) and all of its neighboring polymers (blue). This configuration corresponds to $\mathrm{Pe} = 0$ and $L = 1450\sigma$.

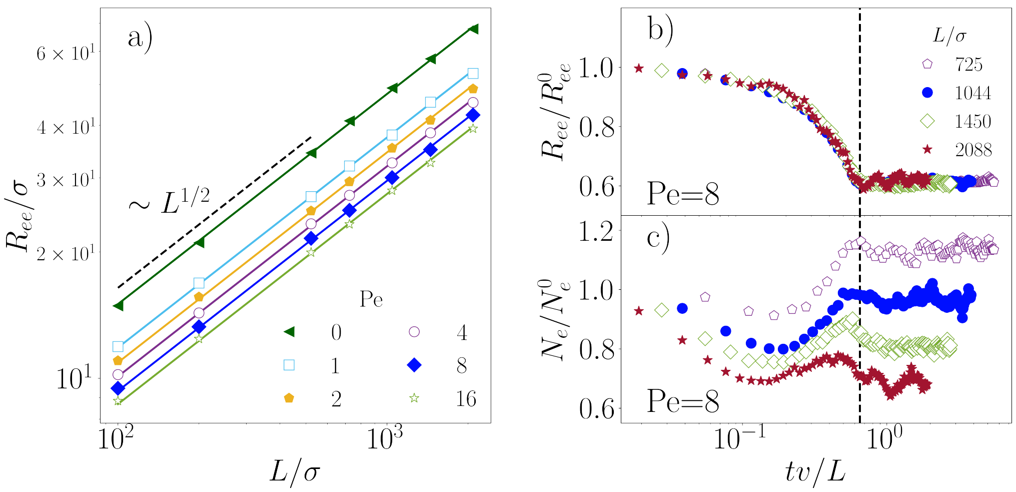

Figure S11. **End-to-end distance and entanglement length across different Péclet numbers.** (a) End-to-end distance R_{ee} as a function of polymer size L/σ for various Péclet numbers Pe, exhibiting the characteristic end-to-end scaling behavior reminiscent of an ideal polymer chain, $\sim L^{1/2}$. (b) R_{ee}/R_{ee}^0 as a function of time for varied L at a fixed $\mathrm{Pe} = 8$, time scaled by L/v. (c) Entanglement length N_e, normalized by the equilibrium value N_e^0 for $\mathrm{Pe} = 8$, as a function of time.

tangled solutions, we investigate polymer solutions with shorter polymer lengths, specifically $L = 25\sigma$. In Fig. S12(a), it becomes evident that the stress plateau is entirely absent from $G(t)$. Instead, the stress relaxation modulus now exhibits a distinct behavior: an initial $\sim t^{-1/2}$ decrease at short times, followed by an eventual exponential decay. This behavior aligns with the predictions of the Rouse model $G(t) \simeq k_B T \rho \, (t/\tau_0)^{-1/2} \, \mathrm{e}^{-t/\tau_R}$ (τ_R is the Rouse time) [9], which describes the relaxation dynamics of polymers in this low-entanglement-regime. This observation confirms that the enhancement of $G(t)$ in entangled systems is indeed due to the altered relaxation mode of entanglements, rather than monomeric friction alone [see Fig. S12(b)].

In contrast to our findings, previous work [13] focuses on passive polymer solutions under uniaxial extension. Under such conditions, polymer chains align with the flow direction, reducing the monomer friction coefficient due to the reduced resistance. The key distinction here is the driving mechanism: in uniaxially deformed passive systems, alignment and stretching reduce friction, whereas in our active system, random self-propulsion leads to enhanced friction through persistent fluctuations.

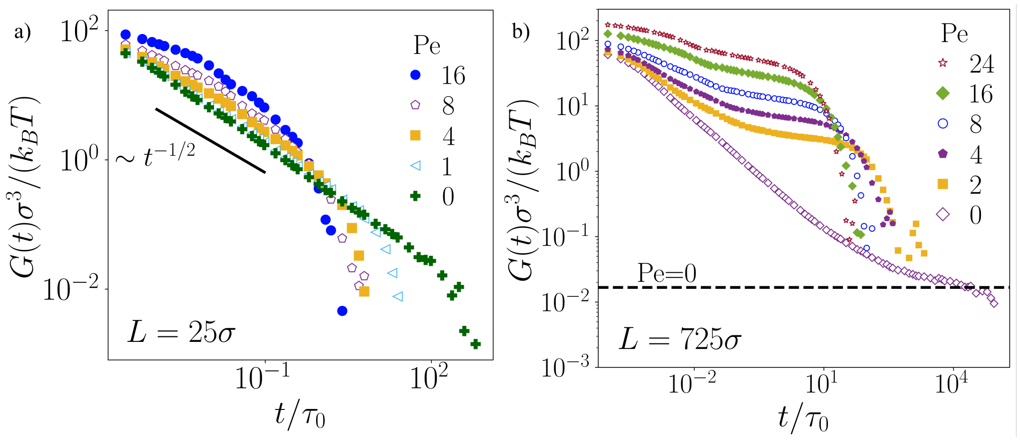

Figure S12. **Stress relaxation contrast between unentangled and highly-entangled active polymers.** (a) Stress relaxation modulus $G(t)$ as a function of t/τ_0 for $L = 25\sigma$ and varying Péclet numbers (unentangled polymers). The stress relaxation modulus exhibits an initial decay characterized by $\sim t^{-1/2}$ behavior, followed by a subsequent exponential decay, notably lacking the entangled plateau. (b) Stress relaxation modulus $G(t)$ as a function of t/τ_0 for $L = 725\sigma$ and varying P'eclet numbers (highly-entangled polymers). The dashed line represents the well-established prediction $G_0 = 4\rho k_B T/(5N_e^0)$.

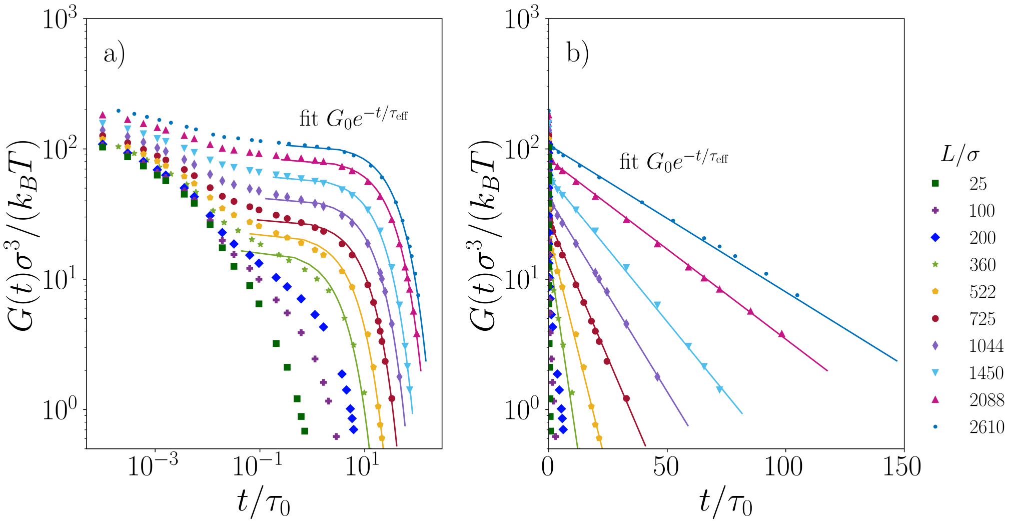

Figure S13. **Stress autocorrelation function for varying polymer lengths at fixed activity.** a) Log-log plot of the stress autocorrelation function, $G(t)$, for different polymer lengths (L) at a fixed activity of $\mathrm{Pe} = 8$, as a function of time. The solid lines represent an exponential fit, $G_0 \exp(-t/\tau_{\rm eff})$, to the simulated data at long times. (b) Semi-logarithmic plot of the same data, with the lines again indicating the exponential fit, $G_0 \exp(-t/\tau_{\rm eff})$.

VIII. Single-mode relaxation of $G(t)$ at long times

The stress autocorrelation function, $G(t)$, can be accurately fitted with a single-mode relaxation function at long times, allowing us to quantitatively extract the terminal relaxation time, $\tau_{\rm eff}$ (see Fig. S13). To compute $G(t)$, we employed the multiple-τ correlator method introduced by Frenkel [14], which is implemented in LAMMPS using the `fix ave/correlate/long` command.

IX. Number of entanglement points and active tube model

While self-propulsion is expected to reduce the number of entanglement points, potentially compromising the validity of the tube model, this effect is most pronounced in systems with relatively few entanglements per polymer chain (e.g., approximately 2 entanglements for $L/\sigma = 100$), as shown in Fig. S14(a). In such cases, even moderate activity

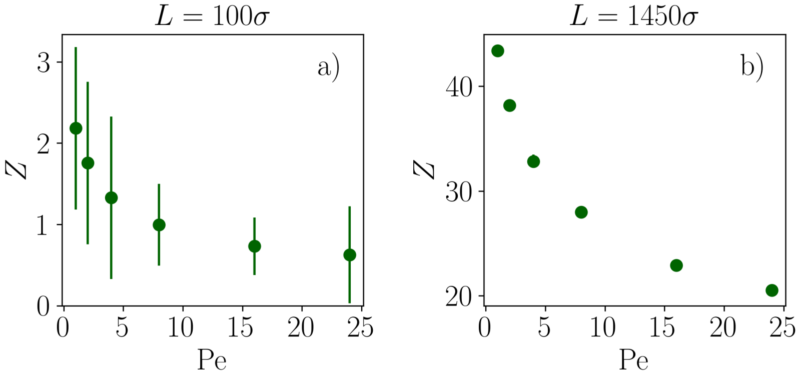

Figure S14. **Entanglement points as a function of activity in low- and highly-entangled polymer solutions.** Number of entanglement points (Z) as a function of the Péclet number (Pe) for (a) loosely entangled polymer solutions with length $L = 100\sigma$ and (b) highly entangled polymer solutions with length $L = 1450\sigma$. Error bars represent the standard deviation of the mean over five independent simulation runs. The plots illustrate how increasing activity (Pe) affects the number of entanglement points in both regimes.

can lead to the loss of these entanglements, rendering the tube model ineffective. This is precisely why our study focuses on highly entangled systems, where each polymer chain initially possesses around 45 entanglement points [see Fig. S14(b)]. Remarkably, even at the highest levels of activity, our simulations reveal that each chain retains approximately 25 entanglement points, demonstrating that significant entanglement persists despite the presence of active forces [see Fig. S14(b)].

Our study introduces a fundamentally new theoretical framework—the *active tube model*—which differs from the classical equilibrium tube model used in passive polymer systems. In passive systems, the characteristic timescale $\tau_e \sim N_e^2$ (where N_e is the entanglement length) governs the relaxation of polymers within a confining tube. However, in our active system, self-propulsion drives each polymer to exit its confining tube on a much shorter timescale $\tau_{\text{eff}} = L/v$. This timescale arises because self-propulsion induces a persistent sliding motion of the polymer, which prevails over random thermal fluctuations and enables the polymer to escape its tube more quickly.

It is important to note that, while the effective tube turnover is rapid (on the timescale τ_{eff}), the system remains highly entangled. Specifically: (a) Upon exiting its tube, an active polymer immediately enters a new tube formed by the surrounding polymers [see Fig. S15]. (b) The tubes themselves are dynamic, continually breaking and reforming on the same timescale $\tau_{\text{eff}} = L/v$.

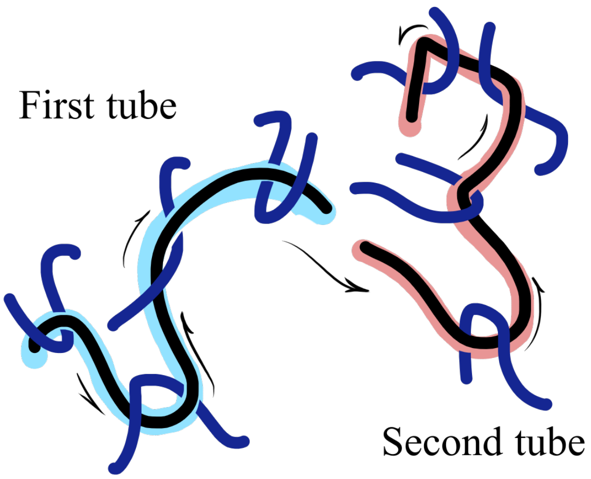

Figure S15. **Dynamic rearrangement of the entangled environment in active polymers.** In the highly-entangled regime, a self-propelled polymer is confined within an effective tube formed by the surrounding self-propelled polymers. Over time, the polymer escapes its initial tube and becomes confined within a newly formed tube, reflecting the dynamic rearrangement of the entangled environment driven by activity.

Our analysis reveals that the system retains a well-entangled structure, with the number of entanglements exceeding 20 for highly entangled active polymers [see Fig. S14(b)]. This observation suggests that the entanglement framework remains robust, even in the presence of rapid self-propelled dynamics. Hence, we use a new model (which could be called an active tube model), not the equilibrium tube model.

[1] M. Han, M. Fruchart, C. Scheibner, S. Vaikuntanathan, J. J. De Pablo, and V. Vitelli, Fluctuating hydrodynamics of chiral active fluids, Nat. Phys. **17**, 1260 (2021).
[2] T. C. O'Connor, N. J. Alvarez, and M. O. Robbins, Relating chain conformations to extensional stress in entangled polymer melts, Phys. Rev. Lett. **121**, 047801 (2018).
[3] N. Koumakis, M. Laurati, S. U. Egelhaaf, J. F. Brady, and G. Petekidis, Yielding of hard-sphere glasses during start-up shear, Phys. Rev. Lett. **108**, 098303 (2012).
[4] I. Svetlizky and Y. Roichman, Spatial crossover between far-from-equilibrium and near-equilibrium dynamics in locally driven suspensions, Phys. Rev. Lett. **127**, 038003 (2021).
[5] H.-M. Chun, Q. Gao, and J. M. Horowitz, Nonequilibrium green-kubo relations for hydrodynamic transport from an equilibrium-like fluctuation-response equality, Phys. Rev. Res. **3**, 043172 (2021).
[6] C. Hargus, J. M. Epstein, and K. K. Mandadapu, Odd diffusivity of chiral random motion, Phys. Rev. Lett. **127**, 178001 (2021).
[7] F. Ghimenti, L. Berthier, G. Szamel, and F. van Wijland, Sampling efficiency of transverse forces in dense liquids, Phys. Rev. Lett. **131**, 257101 (2023).
[8] P. Matus, R. Lier, and P. Surówka, Molecular modeling of odd viscoelastic fluids, Phys. Rev. E **110**, 044605 (2024).
[9] M. Rubinstein and R. H. Colby, Polymer physics, Oxford University Press 10.1002/pi.1472 (2003).
[10] J. D. Dietz and R. S. Hoy, Facile equilibration of well-entangled semiflexible bead–spring polymer melts, J. Chem. Phys. **156**, 014103 (2022).
[11] M. Kröger, J. D. Dietz, R. S. Hoy, and C. Luap, The z1+ package: Shortest multiple disconnected path for the analysis of entanglements in macromolecular systems, Comput. Phys. Commun. **283**, 108567 (2023).
[12] V. Bianco, E. Locatelli, and P. Malgaretti, Globulelike conformation and enhanced diffusion of active polymers, Phys. Rev. Lett. **121**, 217802 (2018).
[13] G. Ianniruberto, Extensional flows of solutions of entangled polymers confirm reduction of friction coefficient, Macromolecules **48**, 6306 (2015).
[14] D. Frenkel and B. Smit, Understanding molecular simulation: from algorithms to applications (Elsevier, 2023).
