# Supplementary material for: Giant activity-induced elasticity in entangled polymer solutions
Source: Nat Commun. 2025 Jun 12;16:5305. doi: 10.1038/s41467-025-60210-9 (PMC12163070; doi:10.1038/s41467-025-60210-9)
Supplement: Supplementary file 2 — Description of Additional Supplementary Files [file 41467_2025_60210_MOESM2_ESM.pdf]

Description of Additional Supplementary Files

File name: Supplementary Movie 1
Description: The movie illustrates the dynamic evolution of primitive paths involving a test polymer (in red) along with its neighboring polymers (in blue) in a simulation setting characterized by Pe=4, L=1450 sigma and rho=0.85.
